# Delayed Antiretroviral Therapy (ART) Initiation among Hospitalized Adults in a Resource-Limited Settings: A Challenge to the Global Target of ART for 90% of HIV-Infected Individuals

**DOI:** 10.1155/2019/1832152

**Published:** 2019-04-01

**Authors:** Prossie Merab Ingabire, Fred Semitala, Moses R. Kamya, Damalie Nakanjako

**Affiliations:** ^1^Department of Medicine, Makerere University College of Health Sciences, Kampala, Uganda; ^2^St. Francis Hospital, Nsambya, Kampala, Uganda; ^3^Infectious Diseases Institute, Makerere University College of Health Sciences, Kampala, Uganda

## Abstract

**Background:**

Combination antiretroviral therapy (cART) initiation in hospital settings, where individuals often present with undiagnosed, untreated, advanced HIV disease, is not well understood.

**Methods:**

A cross-sectional study was conducted to determine a period prevalence of cART initiation within two weeks of eligibility, as determined at hospitalization. Using a pretested and precoded data extraction tool, data on cART initiation status and reason for not initiating cART was collected. Phone calls were made to patients that had left the hospital by the end of the two-week period. Delayed cART initiation was defined as failure to initiate cART within two weeks. Sociodemographic characteristics, WHO clinical stage, CD4 count, cART initiation status, and reasons for delayed cART initiation were extracted and analyzed.

**Results:**

Overall, 386 HIV-infected adults were enrolled, of whom 289/386 (74.9%) had delayed cART initiation, 77/386 (19.9%) initiated cART, and 20/386 (5.2%) were lost-to-follow-up, within two weeks of cART eligibility. Of 289 with delayed ART initiation, 94 (32.5%) died within two weeks of cART eligibility. Patients with a CD4 cell count≥ 50 cells/*μ*l and who resided in ≥8 kilometers from the hospital were more likely to have delayed cART initiation [adjusted odds ratio (AOR) 2.34, 95% CI: 1.33-4.10, p value 0.003; and AOR 1.92, 95% CI: 1.09-3.40, p value 0.025; respectively].

**Conclusion:**

Up to 75% of hospitalized HIV-infected, cART-naïve, cART-eligible patients did not initiate cART and had a 33% pre-ART mortality rate within two weeks of eligibility for cART. Hospital based strategies to hasten cART initiation during hospitalization and electronic patient tracking systems could promote active linkage to HIV treatment programs, to prevent HIV/AIDS-associated mortality in resource-limited settings.

## 1. Background

There is global commitment to fast-track the end of the HIV/AIDS epidemic through the Joint United Nations Programme on HIV/AIDS (UNAIDS) 90-90-90 campaign to test 90% of all people living with HIV, initiate and sustain combination antiretroviral therapy (cART) for 90% of all those diagnosed HIV-infected, and have sustained undetectable viral load among 90% of cART-treated individuals [1]. Access to life saving cART has been achieved by the worlds' most-affected regions of eastern and southern Africa where over 10.3 million people are receiving cART and AIDS-related deaths decreased by 36% since 2010 [2]. Many sub-Saharan African countries, including Uganda, have updated their HIV national guidelines to reflect the World Health Organization's (WHO) Universal Test and Treat guidelines, 2016, to initiate cART as soon as HIV is diagnosed irrespective of CD4 count [3–7]. Recent clinical trial data (HPTN 052) demonstrated that earlier initiation of cART results in near complete interruption of HIV transmission, with a 96% reduction in HIV transmission in sero-discordant couples [8]. However, up to 33% of adults living with HIV and 53% of children living with HIV had not received cART by the end of 2016 [9]. Effectiveness of “test and treat” approaches however remains limited by poor engagement of HIV-infected adults within the national HIV care program.

Consequently, challenges of delayed cART initiation persist in resource-limited settings. Data from cohorts in sub-Saharan Africa showed that most ART-treated adults initiated cART at a median CD4 count range of 87 to 212 cells/*μ*L [10, 11]. Delayed initiation of cART continues to drive morbidity, mortality, and onward transmission of HIV. Causes of late initiation of cART include late diagnosis due to low uptake of HIV testing [10], and limited capacity of clinics to absorb the numbers of all in need of cART, which have been described in ambulatory, community, and observational cohort settings [12–14]. Little has been documented about cART initiation in hospital settings where majority of patients present with advanced HIV disease and opportunistic infections [15, 16], with a potentially high risk of mortality after discharge [17].

In the past, delayed cART initiation has been defined as untreated advanced HIV disease at WHO stage 4 or CD4 ≤200 cells/*μ*l [11, 18, 19]. We defined delayed cART initiation as failure to initiate cART among HIV-infected hospitalized individuals within two weeks of determination of HIV-infection at hospitalization. Our definition of “two weeks” cut-off was based on evidence from the Strategic Timing of Antiretroviral Therapy (START) and Early Initiation of Antiretroviral Therapy for HIV (TEMPRANO) trials which demonstrated profound impact of immediate cART initiation among asymptomatic HIV-infected patients with CD4+ counts 500 cells/*μ*l and over [20–22]. Similarly, the AIDS Clinical Trials Group (ACTG) trials showed remarkable benefit of immediate cART initiation among individuals with advanced disease and opportunistic infections [17, 23]. Our results inform the development of strategies to reach hosptalised HIV-infected adults who are most-at-risk of morbidity and mortality, amidst the wider scale of “test and treat” strategy in many ambulatory HIV care settings.

## 2. Methods

### 2.1. Study Setting

This study was conducted at Mulago hospital, a 1500-bed hospital that serves referred patients from Kampala, the capital city, and outside Kampala. Patients were recruited from three medical wards, where adults with nonsurgical and nonobstetric/gynaecological conditions are admitted. HIV tests and CD4 count measurement for HIV-infected individuals are offered as part of routine medical care. During hospitalization, patients are investigated to obtain confirmatory diagnosis and subsequently treated. HIV infected patients are treated for any opportunistic infection and prepared for cART initiation, except patients with cryptococcal meningitis (CM) in whom cART is deferred for 5 weeks for better outcomes [24]. Upon discharge, cART eligible patients who are not yet ready to initiate cART are routinely given referral notes and appointments to attend the HIV treatment clinic at Mulago or one nearest to their home, depending on the patients' preference. There is no specific follow-up mechanism to determine whether patients honor the referrals or whether they attend HIV clinics different from those indicated on the referral notes.

### 2.2. Study Design and Participants

A cross-sectional study was conducted to determine a period prevalence of cART initiation status within two weeks of diagnosis, as determined by the attending physician during hospitalization. From December 2012 to March 2013, charts of HIV-infected, cART-eligible, cART-naïve patients 13 years and older were consecutively reviewed for cART-initiation status within two weeks of hospitalization. cART initiation status data, CD4 cell count and WHO HIV clinical stage III/IV (determined upon hospitalization at Mulago hosptals' medical wards), were extracted from patients' charts for those still hospitalized for two weeks and more, and through phone calls for those that left hospital within the two-week period. Phone call interviews included preset and precoded questions to determine whether patient was alive, sick/well, had initiated cART, place where cART was initiated (for those that had initiated), and reasons for not initiating (for those that had not initiated). Charts of patients with suspected or confirmed cryptococcal meningitis (CM) were excluded because the Cryptococcal Optimal ART Timing (COAT) trial had shown increased mortality in patients who initiated cART within two weeks of being treated for CM [24].

### 2.3. Measurements

Using a pretested precoded data extraction tool, data extracted included sociodemographic characteristics (age, gender, district of residence, distance (in kilometers) to nearest health center, level of education, occupation, religion, and marital status), and medical history including HIV status, date and place of prior HIV test, use of co-trimoxazole prophylaxis or alternative medicine, prior to hospitalization (for those with known HIV status), previous ambulatory clinic consultations, hospitalization admission in the preceding year, and stage of HIV disease (WHO clinical stage/CD4 count). The main outcome was cART initiation status during hospitalization within two weeks of eligibility (as determined at hospitalization). We also extracted data on date of admission, inpatient diagnosis, CD4 cell count and date of most recent CD4 count whenever available, opportunistic infections in past and present, Karnofsky performance score, comorbidities, and reasons for delayed cART initiation (for those that did not initiate cART during hospitalization). For HIV-infected patients that were no longer in hospital by the end of the two-week period, phone calls were made to them (or the provided next of kin) to ascertain the outcome (dead/alive), cART initiation status, date of cART initiation (for those that had initiated), and reasons for not initiating cART (for those that had not initiated cART). Patients whose calls were picked by neither patient nor the next of kin or other relative provided were considered lost to follow-up.

### 2.4. Ethics Approval and Consent to Participate

Ethical approval was obtained from the School of Medicine Research and Ethics Committee, Makerere University College of Health Sciences. Written informed consent was obtained from all study participants at admission on the medical wards, and assent obtained from the guardian/parent/ next of kin if the patient was below 16 years of age.

### 2.5. Data Analysis

Data was double-entered into EpiData (version 3.1) software, cleaned, and exported to Stata version 12 for analysis. The primary outcome variable was the proportion of hospitalized HIV-infected, cART eligible- cART-naïve, patients who had delayed initiation of cART, defined as failure to initiate cART within two weeks of cART eligibility during hospitalization. Continuous variables such as age, distance to nearest health center, duration of hospitalization, Karnofsky performance status score, and CD4 cell count were summarized using mean and standard deviation, median, and interquartile range. Bivariate analysis using Student's t-test was used for continuous variables that followed a normal distribution. Wilcoxon rank sum test was used for predictors that were not normally distributed. The Chi-square test was used to compare categorical variables among patients with and without delayed cART initiation. Multivariate analysis was used to determine factors associated with delayed cART initiation. Factors with p value < 0.2 after the bivariate analysis were considered for a multivariate analysis. Statistical significance was considered at a p value <0.05. Diagnosis at admission and reasons for not initiating cART were recorded and grouped into frequencies and proportions.

## 3. Results

### 3.1. Sociodemographic and Clinical Characteristics of Hospitalized HIV-Infected Adults

**Figure 1:**
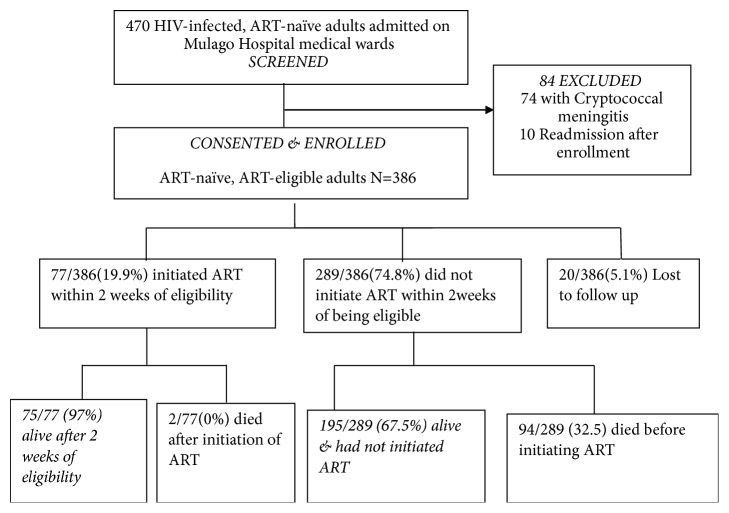
Study profile of HIV-infected adults hospitalized on medical ward at Mulago in December 2012-March 2013.

Between December 2012 and March 2013, a total of 470 HIV-infected, ART-naïve patients were identified and screened for eligibility to participate in the study. Of these, 386 (81.6%) patients were enrolled into the study, and 84 patients were excluded (74 had cryptococcal meningitis and 10 were re-admissions that had already been enrolled), as shown in Figure 1. Of 386 individuals recruited, 193/386 (50.0%) were females, 219/386 (56.7%) were residents of the two neighboring districts of the hospital, 211/386 (54.7%) of the patients had attained primary education, and 384/386 (99.5%) had WHO stage III/IV disease. All HIV-infected adults were eligible for cART initiation, according to the national guidelines at the time. The median distance from home to the nearest health centre was 3 km (IQR 1.6-5km), median age was 32 years (IQR 27-40 years), and median CD4 cell count was 68 (IQR 18-195) cells/*μ*l. Patients who initiated cART had significantly lower CD4 counts than those that delayed, p value 0.005 (Table 1).

### 3.2. Delayed cART Initiation among Hospitalized Patients

**Figure 2:**
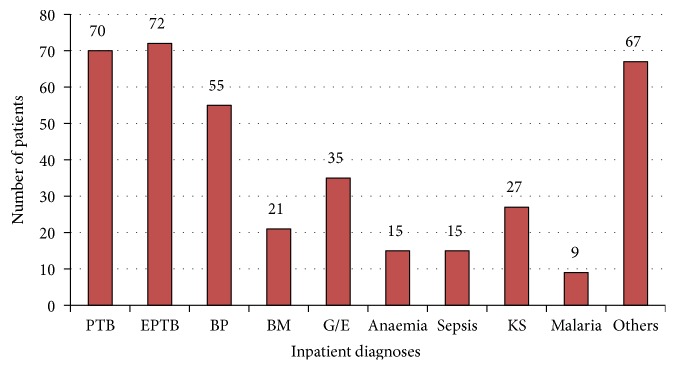
Histogram showing the most common diagnoses among HIV-infected adults hospitalized at Mulago hospital.* PTB, pulmonary tuberculosis; EPTB, extra pulmonary tuberculosis; BP, bronchopneumonia; BM, bacterial meningitis; G/E, gastroenteritis; KS, Kaposis sarcoma, and others included PCP, HIV associated nephropathy, toxoplasmosis, acute hepatitis,* and* recurrent pleural effusions. 74 patients with Cryptococcal meningitis were excluded from the study*.

Overall, 289/386 (74.8%) had delayed cART initiation, since they did not initiate cART within two weeks of eligibility. Only 77/386 (19.9%) initiated cART within two weeks of eligibility, [22/386 (5.7%) initiated during hospitalization, 55/386 (14.2%) initiated after leaving hospital], and 20/386 (5.2%) were lost to follow-up. Of patients with delayed cART initiation, 94/289 (32.5%) died (76 died in hospital and 18 died after discharge) within two weeks of being found cART eligible. The median hospital stay was 8 (IQR 4-13) days, and the commonest causes of hospitalization were tuberculosis (pulmonary and extrapulmonary), followed by bacterial pneumonia (Figure 2). The leading causes of death during hospitalization were tuberculosis [31/94 (33%)], Kaposi's sarcoma 10/94 (11%), and bacterial meningitis 9/94 (10%). The main reason not to initiate cART for the patients that died was refusal of patient/relative to initiate cART, despite doctors' recommendation, because patients had been too sick (as expressed by the relatives).

### 3.3. Poor Linkage to cART before Hospitalization

Prior to the current hospitalization, majority [295/386 (76.4%)] of HIV-infected patients were aware of their HIV-infected status, yet only 132/295 (44.7%) had been enrolled into an HIV care program, yet 291/295 (98.6%) had received cotrimoxazole prophylaxis. This was the first admission within the year for 258/386 (66.8%) patients, although more than half had visited outpatient clinics five times or more during the year prior to this study.

### 3.4. Factors Associated with Delayed ART Initiation

Patients with CD4 counts >50 cells/*μ*l and patients living outside Kampala were more likely to have delayed cART initiation [OR 2.34 (95%CI 1.33-4.10); p value 0.003] and [1.92 (95% CI 1.09-3.40); p value 0.025], respectively (Table 2). The leading reasons for delay of cART initiation, as extracted from patient records and responses to phone interviews, were long period (> two weeks) of preparation of cART 54/195 (27.6%) and failure to honor referral appointments 51/195 (26.1%). Other reasons mentioned were related to patients being too weak to initiate cART (Table 3).

## 4. Discussion

We found a high prevalence of delayed cART initiation among hospitalized patients. Up to 75% of HIV-infected adults did not initiate cART within two weeks of eligibility. Only 20% initiated cART within two weeks, majority (77%) of whom initiated cART after leaving hospital. Of patients with delayed cART initiation, 94/289 (33%) died within two weeks. Up to 80% of patients who died before cART initiation were still in hospital and the main reason for not initiating cART despite physicians' recommendations was postponement (by the patients and relatives) because patients were too sick. Similarly, high mortality was reported among patients that initiated cART during hospitalization in Tanzania, where the main causes of death within the first month of cART were anemia, thrombocytopenia, and severe malnutrition [25]. It remains unclear whether mortality would have been different if cART had been initiated during hospitalization, given that most of the patients that died had Kaposi's sarcoma, tuberculosis, and bacterial meningitis. Important to note too is the role of patients' and relatives' perceptions of the benefits of initiating cART in very sick patients, which was not assessed during this study.

Our results are comparable to previous reports from an HIV testing trial on the same wards where only 62% of surviving HIV-infected participants were linked to HIV care, only 15% received cART, and 35% died, within six months of discharge from hospital [17]. A study in Tanzania showed that less than 1% of HIV-infected patients initiated cART during hospitalization [26]. Despite advances in national guidelines to include the WHO “test and treat” guidelines for initiating cART among HIV-infected adults irrespective of CD4 counts [6], which is largely adhered to in ambulatory clinics and community programs [4], hospital settings continue to present the challenge of advanced untreated HIV disease that is associated with a high postdischarge mortality rate. In addition to scale up the “test and treat” strategy to meet the 90-90-90 targets for 2020, there is a need to fast-track cART initiation among hospitalized patients who continue to present with advanced untreated HIV disease.

Patients with CD4 counts above 50 cells/*μ*l and patients living outside Kampala were more likely to have delayed cART initiation. Similarly, lengthy preparation for cART [54/195 (28%)] and failure to honor referral appointments [51/195 (28%)] were mentioned by individuals that did not initiate cART. Our data from hospitalized patients was comparable to reports from a review of observational cART cohorts in sub-Saharan Africa where patients presented to the health care system with advanced untreated HIV diseases with CD4 cell count <50 cells/*μ*L and WHO stage IV [27]. A recent review of from 27 studies from 18 African countries reported a range of 87-212 cells/*μ*L at cART initiation [10, 11]. Limited access to HIV testing, cART centers, and CD4 counts were previously highlighted as a barrier to timely initiation of cART, particularly in ambulatory HIV/AIDS care settings [28, 29], and indeed increased availability of these services has increased cART initiation rates [17, 30, 31]. Hospital settings, however, present a unique challenge of patients with advanced untreated HIV disease despite large strides that have been taken to test and treat HIV in several ambulatory and community settings [4]. In our study, tuberculosis, Kaposi's sarcoma, and bacterial meningitis were the three leading causes of mortality among HIV-infected adults that did not initiate cART during hospitalization. Delayed diagnosis and treatment of opportunistic infections (OI) continue to delay cART initiation in hospital settings in sub-Saharan Africa [10, 24, 32], although national treatment guidelines encourage concurrent treatment of OI and cART with the exception of cryptococcal meningitis.

We found that 76% of hospitalized HIV-infected patients were aware of their HIV-infection status prior to current hospitalization and had received cotrimoxazole prophylaxis but not cART, and only 45% of them had been enrolled into an HIV care program. Of the patients that did not initiate cART within two weeks of eligibility, 28% were still under preparation for cART, 26% had not honored their referral notes to ART clinics, and another 24% were still considered too weak to initiate cART. Health system challenges such as poor access to diagnostic services, prolonged patient preparation for cART, and limited tracking of referrals continue to affect the quality of health care [17, 28]. In a stepped-wedge cluster randomized trial at lower-level health care facilities in Southwestern Uganda, 38% initiated cART within two weeks after eligibility, although cART initiation rate increased with implementation of an intervention comprising training and coaching of front-line health workers, a point-of-care CD4 cell count testing platform, a revised counselling approach without mandatory multiple preinitiation sessions, and feedback to the facilities on their cART initiation rates [33]. A similar intervention might be useful to support front-line health care providers to handle hospitalized cART eligible individuals, particularly those considered too weak to initiate cART due to ongoing treatment for opportunistic infections. Implementation studies are clearly needed to support the scale-up of already proven interventions of immediate cART for patients, such as immediate initiation of ART among patients receiving anti-tuberculosis treatment [23], and other comorbidities such as bacterial meningitis and Kaposi's sarcoma [34]. There is a need for comprehensive strategies to mitigate the known health system delays to cART initiation, and specific interventions to reach individuals that slip through the ambulatory ART initiation efforts, only to present to hospital settings with untreated advanced HIV disease with a high risk of mortality.

cART initiation was simply by patient self-report, but could not be confirmed via any tracking system. Similarly it was unclear whether patients recorded as “lost to follow-up” had been enrolled into other HIV care facilities in or outside urban Kampala. We recommend development of a national HIV care database with unique identifiers to track patients between different HIV treatment sites. Our definition of delayed cART initiation was limited to two weeks after eligibility, as determined during hospitalization, mainly because of the evidence that early cART initiation within two weeks of eligibility reduced the rate of severe illnesses in clinical trial settings [21, 34].

## 5. Conclusion

Up to 75% of hospitalized HIV-infected, ART-naïve, ART-eligible patients did not initiate cART and had a 33% pre-ART mortality rate within two weeks of eligibility for cART. Hospital based strategies to hasten cART initiation during hospitalization and electronic patient tracking tools could improve linkage to HIV treatment programs and prevent HIV/AIDS-associated mortality in resource-limited settings.

## Figures and Tables

**Table 1:** Characteristics of HIV-infected adults hospitalized at Mulago hospital and their antiretroviral therapy (ART) initiation status within two weeks of eligibility.

Variable	Initiated ART	Delayed ART initiation^∞^	P-value
	N=75	N=195	
	n (%)	n (%)	
*Gender *			0.95
* Female *	37 (49)	97 (49.7)	
* Male*	38 (51)	98 (50.2)	

*Age *			0.51
* ≤ 40 years*	63 (66)	157 (80.5)	
* >40years*	12 (16)	38 (19.5)	

*District of residence Kampala *			0.108
* Kampala*	47 (83)	101 (51.8)	
* Outside*	28 (37)	94 (48.2)	

*Level of education*			0.22
* No formal & Primary *	38 (51)	115 (58.9)	
* Secondary & Tertiary*	37 (49)	80 (41.0)	

*Distance to nearest health Centre*			0.44
* ≤3km*	42 (56)	99 (50.7)	
* >3km*	33 (44)	96 (49.2)	

*Newly diagnosed HIV positive*	60 (80)	161 (82.5)	0.62
*Known HIV positive *	15 (20)	34 (17.4)	

*Disclosure *	60 (80)	149 (76.4)	0.53
*No disclosure*	15 (20)	46 (23.5)	

*Social Support *	72 (96)	182 (93.3)	0.41
*No social support*	3 (4)	13 (6.6)	

*Karnofsky score*			0.15
* ≤40*	66 (85)	157 (80.5)	
* >40*	9 (12)	38 (19.5)	

*¥OP clinics attended in year*			0.15
* <5*	30 (40)	97 (49.7)	
* ≥5*	45 (60)	98 (50.3)	

*WHO HIV clinical stage*			0.07
* stage II&III∗*	30 (40)	97 (49.7)	
* stage IV*	45 (60)	98 (50.1)	

*CD4 cell count*			0.005
* ≤50*	37 (49)	56 (28.7)	
* >50*	38 (51)	126 (64.6)	

∞ Delayed ART initiation referred to failure to initiate ART within two weeks of eligibility, as determined during the current hospitalization.

¥OP clinics, outpatient clinics.

Note: 94 died before ART initiation, 2 died after ART initiation, and 20 were lost to follow-up.

*∗*Only 2/386 patients were stage II.

**Table 2:** Predictors of delayed initiation of cART among HIV-infected adults hospitalized at Mulago hospital.

Effect		Odds ratio	P- value
		OR (95% CI)	
*CD4 cell count/µl *	≤50	1	
	>50	2.34 (1.33 – 4.10)	0.003

*District of residence*	Kampala	1	
	Outside Kampala	1.92 (1.09 – 3.40)	0.025

*Karnofsky Performance status score*	≤40	1	
	>40	1.92 (1.07 – 3.45)	0.67

*Outpatient clinic attendances /year*	<5	1	
	≥5	0.63 (0.35−1.12)	0.11

**Table 3:** Reasons for delayed initiation of cART among HIV-infected cART-naïve adults hospitalized at Mulago hospital.

Reason for not initiating cART in 2 weeks of eligibility	Frequency N=195
	n (%)
*Referral systems *	
Period of preparation for cART initiation >2weeks	54 (27.7)
Did not honor referral date given	51 (26.1)
Review date given more than 2 weeks after discharge	16 (8.2)
Told CD4 above 200mg/*µ*l	9 (4.7)
Attending OPD clinic not integrated with cART	3 (1.5)
Given wrong clinic day	1 (0.5)
*Advanced disease considered too weak to initiate cART*	
Patient readmitted	18 (9.2)
Very sick & being treated for opportunistic infection	18 (9.2)
Very sick and weak to start cART	12 (6.1)
Patient taken to village gave up on life	5 (2.5)
*Patient social support*	
No social support	7 (3.6)
Feared drug reaction	1 (0.5)

## Data Availability

The data used to support the findings of this study are available from the corresponding author upon request.

## Abbreviations

ART: Antiretroviral therapy
cART: Combined antiretroviral therapy
WHO: World Health Organization
START: Strategic Timing of Antiretroviral Therapy
TEMPRANO: Early Initiation of Antiretroviral Therapy for HIV
ACTG: AIDS Clinical Trials Group
CM: Cryptococcal meningitis
COAT: Cryptococcal Optimal ART Timing.
